# Prevalence and Risk Factors of Food Allergy and Its Association With Asthma Coexistence and Severity in Bangladeshi Schoolchildren: A GIS‐Based Cross‐Sectional Study

**DOI:** 10.1002/fsn3.72240

**Published:** 2026-08-19

**Authors:** Nitai Roy, Sultan Mahmud Imran, Md. Fazle Rabbi, Abdullah Al Adib

**Affiliations:** ^1^ Department of Biochemistry and Molecular Biology Patuakhali Science and Technology University Patuakhali Bangladesh; ^2^ Department of Community Health and Hygiene Patuakhali Science and Technology University Patuakhali Bangladesh

**Keywords:** asthma, Bangladesh, food allergy, geospatial analysis, schoolchildren

## Abstract

Food allergies are a critical health issue that increases susceptibility to a range of allergic disorders. However, there is limited understanding of the burden of food allergies in Bangladeshi schoolchildren. This study aimed to determine the prevalence of food allergies and their risk factors among schoolchildren in Bangladesh and to examine the relationship between food allergies and the coexistence and severity of asthma. A cross‐sectional study was conducted among schoolchildren aged 12–18 years, with 828 participants (54.5% girls and 45.5% boys) enrolled in schools across all divisions of Bangladesh. Food allergies and current asthma status were determined based on patients' clinical histories. Associations were examined using Poisson regression with robust variance estimation, yielding adjusted prevalence ratios (aPRs) and 95% confidence intervals (CIs). Among schoolchildren, the 12‐month prevalence of study‐defined food allergies was 29.3% and the prevalence of current asthma was 15.9%. The most commonly reported locally significant offending food allergens were vegetables (76.1%), beef (61.3%), and shellfish (58.0%). Food allergies were more common among girls, older adolescents, lower‐income families, and children residing in Barisal, Sylhet, or Rajshahi. Asthma and food allergies frequently coexist (8.1%), with asthma cases being twice as likely to report food allergies. Food allergies were also associated with higher asthma severity, particularly wheezing and sleep‐disturbing symptoms. GIS mapping revealed marked regional variation, with the greatest coexistence observed in the Barisal and Sylhet divisions. This study demonstrated the significant burden of food allergies and their frequent coexistence with asthma among Bangladeshi school children. Targeted awareness campaigns, early diagnosis, and region‐specific interventions are critical for reducing the dual burden of food allergies and asthma, particularly in high‐prevalence areas, such as Barisal and Sylhet.

## Graphical abstract

This nationwide study revealed a high prevalence of food allergies (29.3%) and asthma (15.9%) among Bangladeshi schoolchildren, with frequent coexistence and greater severity in certain regions highlighting the urgent need for targeted awareness, early diagnosis, and region‐specific interventions.

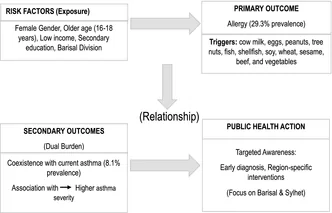

## 1 Introduction

Food allergies represent a growing public health concern, particularly among children, as they not only pose a risk of adverse physical reactions but also contribute to significant physical and psychological strain on their overall health and quality of life. These conditions result from an adverse immune response to specific food proteins and currently affect approximately 6% of children worldwide, increasing to 8% in Western countries, highlighting a trend that parallels the increasing prevalence of other allergic disorders (Sicherer and Sampson 2006; Cianferoni and Spergel 2009). This increasing trend is particularly concerning in children, whose underdeveloped immune systems make them more vulnerable to food allergens in early life (Children's Hospital Los Angeles 2016).

In particular, dysfunction of the innate immune system during early life may play a critical role in the development and prediction of persistent food allergies in children (Neeland et al. 2017). The early years of life are crucial for physical growth and neurodevelopment; however, many children begin to experience allergic reactions to food during this period (Bock 1987). A systematic review suggested that children with food allergies experience greater challenges, including reduced physical functioning, poorer mental and general health, increased bodily pain, and diminished emotional, social, and psychological quality of life (Morou et al. 2014).

In clinical terms, children with food allergies may experience a wide range of symptoms, ranging from mild reactions such as redness, itchy rashes (hives), and eczema to severe manifestations, including anaphylaxis and asthma‐like symptoms, such as coughing, nasal congestion, wheezing, and breathing difficulties (Food Allergies in Children 2025). Alarmingly, a study found that food‐induced anaphylaxis is the primary trigger in children, contributing up to 85% of cases (De Silva et al. 2008). Studies from countries such as Australia have indicated that peanuts (18%) and cashew nuts (13%) are primary triggers of anaphylaxis in children (De Silva et al. 2008). Another study conducted in the United States suggested that milk, eggs, and peanuts were the most common foods that triggered allergic reactions in children (Fleischer et al. 2012).

In Bangladesh, the most commonly reported allergenic foods include eggs, milk (including lactose), peanuts, soybeans, fish, crustacean shellfish, sesame seeds, celery, mustard, gluten‐containing grains (such as wheat, barley, and rye), tree nuts (such as hazelnuts, almonds, and walnuts), and food products with sulfite levels ≥ 10 mg/kg or higher (Bangladesh Food Safety Authority 2017). Despite the availability of this list, there is currently no cure for food allergies. Thus, strict avoidance remains the only preventive strategy, although it is often difficult to implement in daily life, particularly in school‐aged children.

Food allergies rarely occur when isolated from other allergic diseases. They are closely associated with asthma, which is the most common chronic condition in children and a major global noncommunicable disease. The World Health Organization (WHO) has reported that asthma remains underdiagnosed and undertreated, particularly in low‐ and middle‐income countries such as Bangladesh (World Health Organization 2024). Asthma and food allergies have a clinical relationship. Food allergens may induce asthma symptoms in susceptible children and, in turn, contribute to the risk and intensity of allergic reactions, including anaphylaxis (Caffarelli et al. 2016). This association is supported by shared immunopathological mechanisms. Both food allergy and allergic asthma are driven by a type‐2 helper T‐cell (Th2)– and IgE‐mediated immune response, in which exposure to allergens induces IL‐4/IL‐13‐dependent class switching of B cells to produce allergen‐specific IgE (Caminati et al. 2018; Gao et al. 2025). This IgE binds high‐affinity FcεRI receptors on mast cells and basophils in the airways, skin and gut; subsequent re‐exposure to the food allergen leads to cross‐linking of IgE and rapid degranulation, releasing histamine, leukotrienes and other mediators that cause bronchoconstriction, airway inflammation and, in severe cases, anaphylaxis (Nguyen et al. 2021; Ogulur et al. 2025; Vitte et al. 2022). This shared Th2/IgE pathway and impaired epithelial barrier function help explain why food allergy and asthma frequently coexist as part of the “atopic march” and why children with food allergy have an increased risk of developing asthma and experiencing more severe, sometimes life‐threatening reactions when both conditions are present (Gao et al. 2025; Ogulur et al. 2025; Vitte et al. 2022).

This coexistence has been investigated in many international studies, and the results have continued to indicate that children with asthma have an increased likelihood of developing food allergies, and vice versa (Cherian et al. 2022). The dual burden is a potential significant factor affecting the daily functioning of children, their attendance at school, and psychological maturation (Hassan et al. 2002; Pate et al. 2022; Sansweet et al. 2024). However, no studies have examined the prevalence of food allergies and how they relate to asthma, although there are an estimated 4 million children who experience asthma symptoms in Bangladesh. This lack of evidence does not allow for the formulation of comprehensive intervention programs for Bangladeshi children. The prevalence of asthma has been a significant morbidity issue in Bangladesh, and its prevalence rates in the country have been fairly stable, at approximately 7% of the population, over a decade, with recent epidemiological data on the prevalence being rather scarce (Rakhshanda et al. 2023). Although it is estimated that 4 million children in Bangladesh experience asthma symptoms, there seems to be a dearth of studies on the prevalence of food allergies and their association with asthma (Hassan et al. 2002). This lack of evidence can hinder the development of holistic intervention programs to address children in Bangladesh.

Despite increasing concern, no large‐scale epidemiological studies have investigated food allergies and their correlation with childhood asthma in Bangladesh. Food allergies in school‐aged children frequently coexist with other allergic conditions, notably asthma; however, they are often underrecognized and insufficiently documented. Given this critical knowledge gap, the present cross‐sectional study was conducted to estimate the prevalence of food allergies and assess their relationship with the presence and severity of asthma among schoolchildren in Bangladesh. Geographic Information System (GIS) technology was utilized to pinpoint spatial distribution patterns, clusters, and areas with a high coexistence burden of food allergy and asthma. The goal of this spatial analysis is to generate evidence that is specific to each region so that health interventions and public health strategies can be better planned. The study aims to provide significant insights to improve clinical management, inform policy formulation, and reinforce educational initiatives focused on protecting the health and welfare of schoolchildren in Bangladesh.

## 2 Methodology

### 2.1 Study Area

Bangladesh is a densely populated country located in northeastern South Asia, bordered by India to the west, north, and east; Myanmar to the southeast; and the Bay of Bengal to the south, covering an area of approximately 147,570 km^2^. Bangladesh's administrative structure is organized into eight divisions: Dhaka, Chattogram (Chittagong), Khulna, Rajshahi, Barisal (Barisal), Sylhet, Rangpur, and Mymensingh. A cross‐sectional survey on food allergies and their association with the coexistence and severity of asthma was conducted among schoolchildren across all divisions in Bangladesh from December 2023 to April 2024.

### 2.2 Study Subjects, Design and Sampling

This cross‐sectional study enrolled schoolchildren aged 12–18 years from Bangladeshi public institutions. Students younger than 12 years or older than 18 years were excluded because the study focused on adolescents in secondary and higher secondary education who are developmentally and educationally distinct from younger children. First, a complete list of districts within each division was obtained from the Bangladesh Bureau of Statistics. Within each division, one district was selected using computer‐generated simple random sampling, ensuring that every district in a division had an equal probability of selection. Second, a list of all eligible secondary and higher secondary schools and colleges in each selected district was obtained from the district education office. From this list, two schools per district were selected using the same computer‐generated simple random sampling method. Pre‐primary and primary institutions were not included. Third, a list of eligible students from each school (the sampling frame) was obtained, and simple random sampling was used to select those who would participate. Only secondary and higher secondary schools and colleges were included in the sampling process. Pre‐primary and primary institutions were not included in this study. The final sample consisted of 828 students from 16 schools (two per division). Most participants were from rural areas, whereas the remainder were from urban areas.

The required sample size was calculated using the formula *n* = *z* ^2^ *p*(1 − *p*)/*d* ^2^, where *p* is the estimated population proportion (0.50), d is the allowable margin of error (0.05), *z* is the *z*‐score corresponding to the 95% confidence interval (1.96), and *n* is the required sample size. A design effect of 2.0 was applied to account for clustering in the multistage cluster sampling procedure. This resulted in a sample size of 768 participants (384 × 2.0). The final sample size was increased to 828 schoolchildren to improve statistical power, ensure subgroup representativeness, and accommodate potential nonresponses.

### 2.3 Definitions

#### 2.3.1 Food Allergy

Food allergy status over the past 12 months was assessed using symptom‐based diagnostic criteria that have been previously developed and validated in epidemiologic food allergy research, combining a convincing clinical history with organ‐specific symptoms and temporal relationship to food intake, which represented their current food allergy status (Boyce et al. 2010; Grabenhenrich et al. 2016; Arshad et al. 2014; Venkataraman et al. 2014, 2018). The criteria were aligned with international guideline recommendations that emphasize an allergy‐focused history and symptom pattern when oral food challenges are not feasible at scale (Boyce et al. 2010; Muraro et al. 2022; Sicherer and Sampson 2017; Ziyab 2019). The predefined criteria for food allergy diagnosis included the following components:

1. Reported exposure and reaction: Participants reported at least one adverse reaction to a common food allergen, including cow milk, eggs, peanuts, tree nuts, fish, shellfish, soy, wheat, sesame, beef, and vegetables (Sicherer and Sampson 2017) (both globally common foods and those identified as culturally or clinically relevant in Bangladesh, such as locally consumed meat and vegetables). To reduce misclassification, the survey included probes designed to screen out nonallergic reactions, such as lactose intolerance (specific for milk), by distinguishing systemic (hives, wheezing) from isolated gastrointestinal symptoms; this approach has been shown to improve the specificity of questionnaire‐based food allergy classification (Boyce et al. 2010; Grabenhenrich et al. 2016; Sicherer and Sampson 2017).
2. Recognized allergic symptoms: At least one recognized allergic symptom, including the following:
  - Localized symptoms: itching, sting/burning of the lips/mouth or throat, urticaria/hives, and angioedema.
  - abdominal symptoms: nausea, vomiting, crampy/colicky abdominal pain, diarrhea;
  - Respiratory symptoms: wheezing, stridor, watery rhinitis, and redness of the eyes or nose.
  - Skin symptoms: urticaria, itching, flushed skin, and worsening eczema; e. Systemic reactions: anaphylaxis, syncope, and
3. Temporal relationship: Onset of symptoms within 2 h of food ingestion.

Children were classified as having food allergy if they met criteria 1, 2, and 3. This method enabled us to determine the 12‐month prevalence of food allergy, referred to throughout the paper as “study‐defined food allergy,” conceptually similar to “symptom‐convincing” food allergy used in validated international surveys (Grabenhenrich et al. 2016; Ziyab 2019).

#### 2.3.2 Asthma

Current asthma (asthma within the past 12 months) was identified based on affirmative responses to the following questions: “history of physician‐diagnosed asthma,” “wheezing in the past 12 months,” and/or “asthma treatment in the past 12 months (Silva et al. 2019; Ziyab 2019).” The severity of asthma symptoms over the past year was evaluated by assessing the frequency of wheezing episodes, wheezing severe enough to limit speech to one or two words between breaths and wheezing that disrupted sleep, following validated severity domains used in international questionnaires and cohort studies (Silva et al. 2019; Ziyab 2019).

### 2.4 Survey Method and Management

All children aged 12–18 years from each selected school were invited to participate. Data were collected through structured questionnaires administered through both self‐completion in classrooms and face‐to‐face interviews, depending on respondents' literacy levels and preferences. The data collection process was conducted by a team of five trained interviewers who received comprehensive training on data collection techniques, ethical considerations, and respondent interactions. The questionnaire was originally created in English and then translated into Bangla through a forward and backward translation process to ensure clarity, accuracy, and cultural relevance. A pilot test was conducted to assess the reliability of the translated version prior to data collection. The importance of maintaining confidentiality and assuring participants that their identities would remain protected in all reports was clearly communicated to the students. Permission was obtained from the heads of the institutions before conducting the survey. The surveys were administered during classes and breaks, with additional permission obtained from the respective class teachers to minimize disruption of academic activities. Only students who were present in class during the survey were included, and data were exclusively collected from them. For some information, such as how the baby was delivered, whether they breastfed, or their demographic background, parents or guardians were contacted directly via telephone. Questionnaires with missing, inconsistent, or unclear responses were returned to the respondents for clarification and completion. The class teachers and investigators reviewed the completed questionnaires to minimize errors and enhance the reliability of the dataset.

### 2.5 Study Variables

The independent variables in this study included sociodemographic and clinical factors. Student‐reported variables were age, sex, residential area, level of education, family size, home division, and knowledge of food allergies. To avoid misclassification for items that adolescents might not know accurately, selected variables were obtained from parents or guardians. Specifically, mode of delivery, history of breastfeeding, monthly family income, and parental history of food allergies, parents or guardians were collected through structured telephone interviews with parents/guardians. These procedures were used to improve accuracy and minimize recall bias for parental and early‐life variables. The dependent variable was the presence of study‐defined food allergy.

### 2.6 Ethical Consideration

This study was conducted in accordance with the ethical principles of the Declaration of Helsinki. Ethical approval was obtained from the Institutional Ethics Committee of Patuakhali Science and Technology University (approval no. PSTU/IEC/2023/65 (5)). Prior to participation, all students were provided with a comprehensive explanation of the study's objectives, procedures, potential risks, and benefits. Written informed consent was obtained from the participants and their legal guardians (where applicable) before participating in the study. Participation in the study was voluntary, and the students were advised that they could withdraw at any time without any consequences. Rigorous steps were taken throughout the study to ensure the confidentiality and anonymity of the collected data.

### 2.7 Statistical Methods

Completed questionnaires were checked, coded, and entered into IBM SPSS Statistics v27.0.1 for analysis. Descriptive statistics were computed to summarize the sociodemographic and clinical characteristics of the study population. The 12‐month prevalence of food allergies was estimated using 95% confidence intervals (CIs). Poisson regression with robust variance was used to examine associations. In the first stage, food allergy was modeled as the dependent variable, with sociodemographic and clinical characteristics as independent variables, producing prevalence ratios (PRs) with 95% confidence intervals (CIs). In the second stage, separate models were fitted to assess the association between food allergies and asthma outcomes including coexistence and severity. ArcGIS 10.8 was used for spatial analysis across the divisions in terms of food allergy, asthma, and their coexistence. The maps were created using shapefiles received from official government mapping websites, which are freely available to the public. Statistical significance was set at *p* < 0.05.

## 3 Results

### 3.1 Characteristics of the Study Population

A total of 828 valid responses were analyzed, including 377 boys (45.5%) and 451 girls (54.5%). Most of the participants were from rural areas (732, 88.4%). Most of the participants were aged 12–13 years (377, 45.5%). More than half of the students (428, 51.7%) came from families with a monthly income ≤ 15,000. The majority of the students were at the secondary level of education (85%) and were born through normal deliveries (83.3%). Almost all participants had breastfeeding experience (99.4%) and were aware of food allergies (97.1% prevalence). More than half of the participants (52.5%) had no parental history of food allergies. The highest percentage of participants in this study was from the Sylhet division (14.9%), whereas the lowest percentage was from the Khulna division (10.1%) (Table 1).

**TABLE 1:** Socio‐demographic characteristics of the study sample (*N* = 828).

Variables	Categories	Frequency	Percentages (%)	Data source
Gender	Female	451	54.5%	Child
	Male	377	45.5%	
Age (years) [14.21 ± 1.913]	12–13	377	45.5%	Child
	14–15	257	31.0%	
	16–18	194	23.4%	
Residence	Rural	732	88.4%	Child
	Urban	96	11.6%	
Monthly family income (Bangladeshi Taka, BDT)	≤ 15,000	428	51.7%	Parent/Guardian
	> 15,000	400	48.3%	
Home division	Dhaka	100	12.1%	Child
	Rajshahi	98	11.8%	
	Rangpur	107	12.9%	
	Mymensingh	108	13.0%	
	Sylhet	123	14.9%	
	Khulna	84	10.1%	
	Chattogram	110	13.3%	
	Barisal	98	11.8%	
Education level	Secondary	704	85.0%	Child
	Higher‐secondary	124	15.0%	
Family size	1–2	21	2.5%	Child
	3–5	603	72.8%	
	≥ 6	204	24.6%	
Mode of delivery	Normal	690	83.3%	Parent/Guardian
	Cesarean	138	16.7%	
Breastfeed ever	No	5	0.6%	Parent/Guardian
	Yes	823	99.4%	
Knowledge of food allergy	No	24	2.9%	Child
	Yes	804	97.1%	
Parental history of food allergy	No	435	52.5%	Parent/Guardian
	Yes	393	47.5%	

*Note:* Analysis: Descriptive statistics were used to summarize the characteristics of the study participants.

### 3.2 Common Food Allergen Reported by the Participants

**FIGURE 1:**
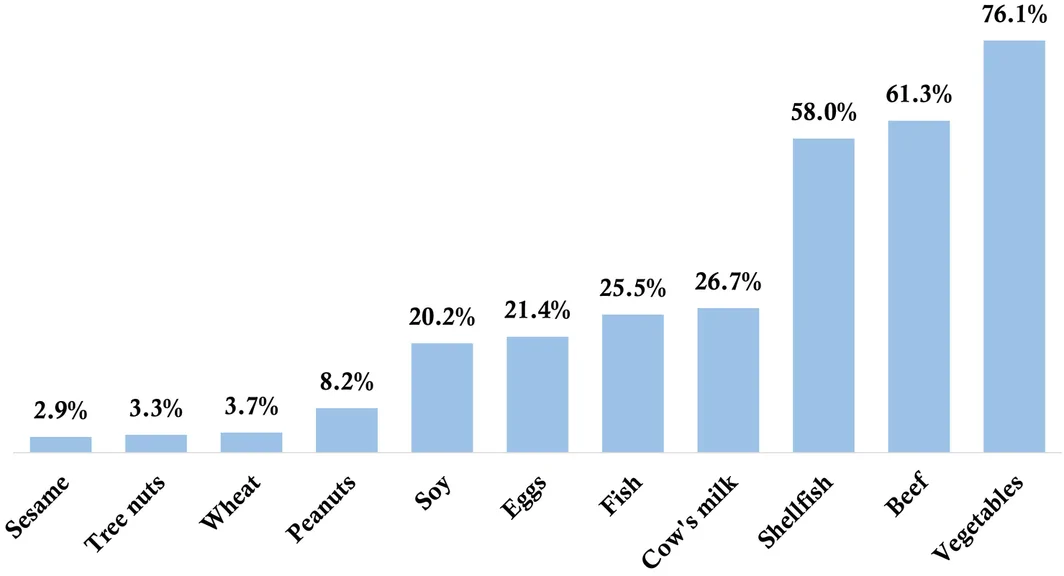
Common food allergen among children with current food allergy.

According to the study participants, vegetables (76.1%), beef (61.3%), and shellfish (58.0%) were the most common foods that caused allergies. Based on their reports, sesame (2.9%), tree nuts (3.3%), and wheat (3.7%) are the least common foods causing food allergies (Figure 1).

### 3.3 Reactions to Foods

Of the 828 participants in this study, 243 were identified to have study‐defined food allergies. Among these participants, the majority (80.6%) experienced skin symptoms such as itching, stinging, or burning of the lips/mouth or throat, urticaria (hives), and flushing. A small proportion of participants with a study‐defined food allergy reported abdominal symptoms, including nausea (22.2%), vomiting (11.5%), and abdominal pain (31.7%). Respiratory symptoms were observed among the participants: 16.9% experienced wheezing, 26.3% reported stridor (noisy or difficult breathing), 32.9% experienced watery rhinitis, and 39.5% experienced redness of the eyes or nose. Anaphylaxis, a severe and potentially life‐threatening condition, was identified in 22.2% of students diagnosed with study‐defined food allergies (Table 2).

**TABLE 2:** Reactions to foods according to the affected organ system among children with study‐defined food allergy.

Reaction	Presence of symptoms with study‐defined food allergy (*n* = 243)		Presence of symptoms without study‐defined food allergy (*n* = 585)		Children without the symptom	
	Frequency	%	Frequency	%	Frequency	%
Skin symptoms						
Itching, sting/burning of the lips/mouth or throat, urticaria/hives, flushing	196	80.6	106	18.1	526	63.5
Angioedema	91	37.4	34	5.8	703	84.9
Worsening eczema	46	18.9	20	3.4	762	92.0
Abdominal symptoms						
Nausea	54	22.2	32	5.5	742	89.6
Vomiting	28	11.5	11	1.9	789	95.3
Crampy/colicky abdominal pain	77	31.7	37	6.3	714	86.2
Diarrhea	11	4.5	5	0.9	812	98.1
Respiratory symptoms						
Wheeze	41	16.9	21	3.6	766	92.5
Stridor (noisy or trouble breathing)	64	26.3	23	3.9	741	89.5
Watery rhinitis	80	32.9	39	6.7	709	85.6
Redness of eyes/nose	96	39.5	37	6.3	694	83.9
Systemic reaction						
Anaphylaxis	54	22.2	23	3.9	751	90.7
Syncope (fainting)	11	4.5	3	0.5	814	98.3

*Note:* Analysis: Descriptive statistics (frequency and percentage) were used to summarize reactions to foods according to the affected organ system.

### 3.4 Prevalence of Study‐Defined Food Allergy and Current Asthma

**FIGURE 2:**
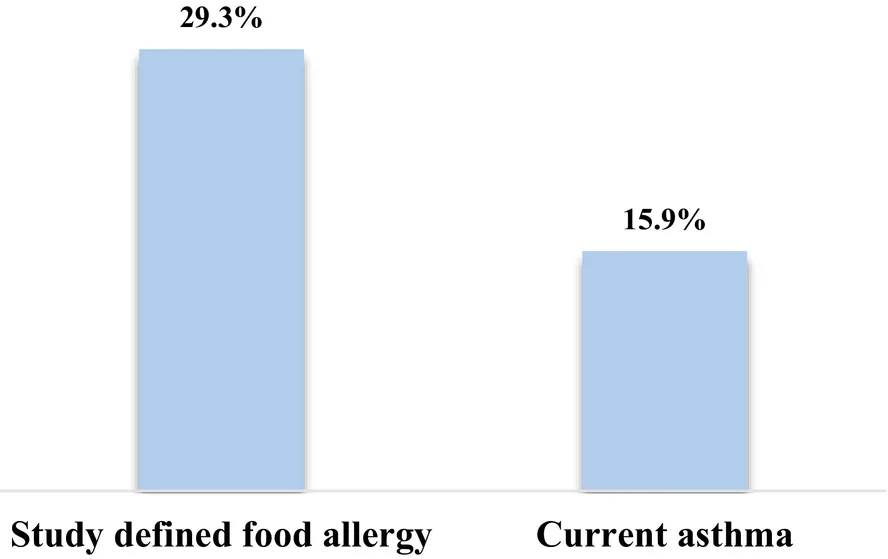
Prevalence of study defined food allergy and current asthma among schoolchildren.

The 12‐month prevalence of study‐defined food allergies was estimated to be 29.3% (243/828), whereas the past 12‐month prevalence of current asthma among schoolchildren was 15.9% (132/828) (Figure 2). Based on sex, 20.4% of male participants had a study‐defined food allergy compared to 36.5% of female participants, indicating a higher prevalence among females. Similarly, current asthma was identified in 17.5% of male children, while 14.6% of female children were affected (Table 3).

**TABLE 3:** Prevalence of study‐defined food allergy and current asthma.

		Study‐defined food allergy	Current asthma
Total analytical sample	*n*/total	243/828	132/828
	Prevalence %	29.3	15.9
Male	*n*/total	77/377	66/377
	Prevalence %	20.4	17.5
Female	*n*/total	166/451	66/451
	Prevalence %	36.8	14.6
*p*‐value		< 0.001	0.294

*Note:* Analysis: Differences in prevalence between males and females were assessed using the chi‐square test.

### 3.5 Factors Associated With Study‐Defined Food Allergy

The associations between various factors and food allergies were analyzed (Table 4). The study revealed that female participants were 1.82 times more likely (aPR =1.82, 95% CI: 1.40–2.35) to have a study‐defined food allergy than male participants, placing them at a higher risk of food allergies. The study‐defined food allergy was less prevalent among 12–13‐year‐old school children (aPR = 0.45, 95% CI: 0.32–0.65) compared to 16–18‐year‐old school children. In addition, low monthly family income (≤ 15,000), secondary education level, and lack of knowledge about food allergies were associated with an increased prevalence of study‐defined food allergies in these children (aPR = 1.29, 95% CI: 1.03–1.61; aPR = 2.09, 95% CI: 1.37–3.18; and aPR = 1.59, 95% CI: 1.02–2.47, respectively). Children in the Barisal division were more likely to have defined food allergies than those in Dhaka (aPR = 0.67, 95% CI: 0.45–1.00), Rangpur (aPR = 0.37, 95% CI: 0.22–0.62), Mymensingh (aPR = 0.42, 95% CI: 0.28–0.63), Khulna (aPR = 0.67, 95% CI: 0.49–0.92), and Chattogram (aPR = 0.60, 95% CI: 0.42–0.88).

**TABLE 4:** Crude and adjusted associations between various factors and study‐defined food allergy.

Variables	Study‐defined food allergy % (*n*/total)	Crude PR	95% CI		Adjusted PR^a^	95% CI		*p*
			LL	UL		LL	UL	
**Gender**								
Female	36.8 (166/451)	1.80	1.43	2.27	1.82	1.40	2.35	< 0.001
Male	20.4 (77/377)	1.00			1.00			
**Age (years)**								
12–13	21.2 (80/377)	0.71	0.53	0.95	0.45	0.32	0.65	< 0.001
14–15	40.9 (105/257)	1.37	1.05	1.77	0.73	0.50	1.05	0.085
16–18	29.9 (58/194)	1.00			1.00			
**Living area**								
Rural	29.4 (215/732)	1.01	0.72	1.40	0.85	0.61	1.18	0.330
Urban	29.2 (28/96)	1.00			1.00			
**Family monthly income (Bangladeshi Taka, BDT)**								
≤ 15,000	32.2 (138/428)	1.23	0.99	1.52	1.29	1.03	1.61	0.024
> 15,000	26.3 (105/400)	1.00			1.00			
**Home Division**								
Dhaka	23.0 (23/100)	0.52	0.34	0.80	0.67	0.45	1.00	0.048
Rajshahi	39.8 (39/98)	0.91	0.65	1.26	0.94	0.69	1.30	0.719
Rangpur	15.9 (17/107)	0.36	0.22	0.59	0.37	0.22	0.62	< 0.001
Mymensingh	21.3 (23/108)	0.49	0.32	0.74	0.42	0.28	0.63	< 0.001
Sylhet	30.9 (38/123)	0.70	0.50	1.00	0.84	0.57	1.23	0.380
Khulna	36.9 (31/84)	0.84	0.59	1.20	0.67	0.49	0.92	0.012
Chattogram	26.4 (29/110)	0.60	0.41	0.88	0.60	0.42	0.88	0.008
Barisal	43.9 (43/98)	1.00			1.00			
**Education Level**								
Secondary	30.1 (212/704)	1.20	0.87	1.67	2.09	1.37	3.18	0.001
Higher‐secondary	25.0 (31/124)	1.00			1.00			
**Family members**								
1–2	23.8 (5/21)	0.97	0.44	2.16	0.93	0.50	1.74	0.829
3–5	31.2 (188/603)	1.27	0.97	1.66	1.25	0.99	1.60	0.064
6 or above	24.5 (50/204)	1.00			1.00			
**Mode of delivery**								
Normal Delivery	28.0 (193/690)	0.77	0.60	0.99	0.86	0.67	1.11	0.240
Cesarean birth	36.2 (50/138)	1.00			1.00			
**Breastfeeding ever**								
No	40.0 (2/5)	1.37	0.46	4.02	1.96	0.78	4.90	0.152
Yes	29.3 (241/823)	1.00			1.00			
**Knowledge about food allergy**								
No	50.0 (12/24)	1.74	1.15	2.63	1.59	1.02	2.47	0.039
Yes	28.7 (231/804)	1.00			1.00			
**Parental history of food allergy**								
No	17.7 (77/435)	0.42	0.33	0.53	0.46	0.36	0.59	< 0.001
Yes	42.2 (166/393)	1.00			1.00			

*Note:* Analysis: Crude and adjusted prevalence ratios were estimated using Poisson regression with robust variance.

Abbreviations: CI, confidence interval; PR, prevalence ratio.

^a^ Variables which are in the crude model were simultaneously included in the adjusted (multivariable) model.

### 3.6 Study Defined Food Allergy and Coexistence of Current Asthma

The relationship between study‐defined food allergy and the coexistence of asthma was evaluated (Table 5). Among the participants with current asthma (*n* = 132), 67 had food allergies. Participants with current asthma were twice as likely to have a defined food allergy than those without asthma.

**TABLE 5:** Associations of study‐defined food allergy with the coexistence and severity of current asthma.

Variables	Category	Total, *n* (%)	Study‐defined food allergy	Study‐defined food allergy	Adjusted PR	95% CI (LL–UL)
			Yes, *n* (%)	No, *n* (%)		
Asthma status	Current asthma	132 (15.9)	67 (27.6)	65 (11.1)	2.01	1.62–2.48
	None	696 (84.1)	176 (72.4)	520 (88.9)	1.00	
Wheezing attacks in the past 12 months	1–3	88 (10.6)	50 (74.6)	38 (58.5)	0.99	0.65–1.53
	≥ 4	44 (5.3)	17 (25.4)	27 (41.5)	1.00	
Wheezing that limited speech in the past 12 months	No	62 (7.5)	27 (40.3)	35 (53.8)	0.62	0.43–0.87
	Yes	70 (8.5)	40 (59.7)	30 (46.2)	1.00	
Wheezing that disturbed sleeping in the past 12 months	1 night per week	59 (7.1)	31 (46.3)	28 (43.1)	0.56	0.40–0.79
	> 1 nights per week	42 (5.1)	12 (17.9)	30 (46.2)	0.31	0.19–0.52
	Never	31 (3.7)	24 (35.8)	7 (10.8)	1.00	

*Note:* Analysis: Adjusted prevalence ratios were estimated using multivariable Poisson regression with robust variance.

Abbreviations: LL, lower limit; PR, prevalence ratio; UL, upper limit.

### 3.7 Study Defined Food Allergy and the Severity of Symptoms of Current Asthma

Table 5 shows the relationship between study‐defined food allergy and the severity of asthma symptoms. Specifically, the study defined food allergy as less common among schoolchildren who experienced one to three wheezing attacks in the past 12 months (aPR = 0.99, 95% CI: 0.65–1.53) compared with those who experienced ≥ 4 wheezing attacks during the same period. Additionally, the study defined food allergy as less prevalent in schoolchildren whose wheezing was not severe enough to limit speech (aPR = 0.62, 95% CI: 0.43–0.87) than in those whose wheezing did limit speech in the past 12 months. Furthermore, the study defined food allergy as less frequent in schoolchildren who experienced wheezing that disrupted sleep either once per week (aPR = 0.56, 95% CI: 0.40–0.79) or > 1 night per week (aPR = 0.31, 95% CI: 0.19–0.52) compared with those who never experienced wheezing that disturbed sleep in the previous 12 months.

### 3.8 GIS‐Based Distribution of Study‐Defined Food Allergy Across Divisions

**FIGURE 3:**
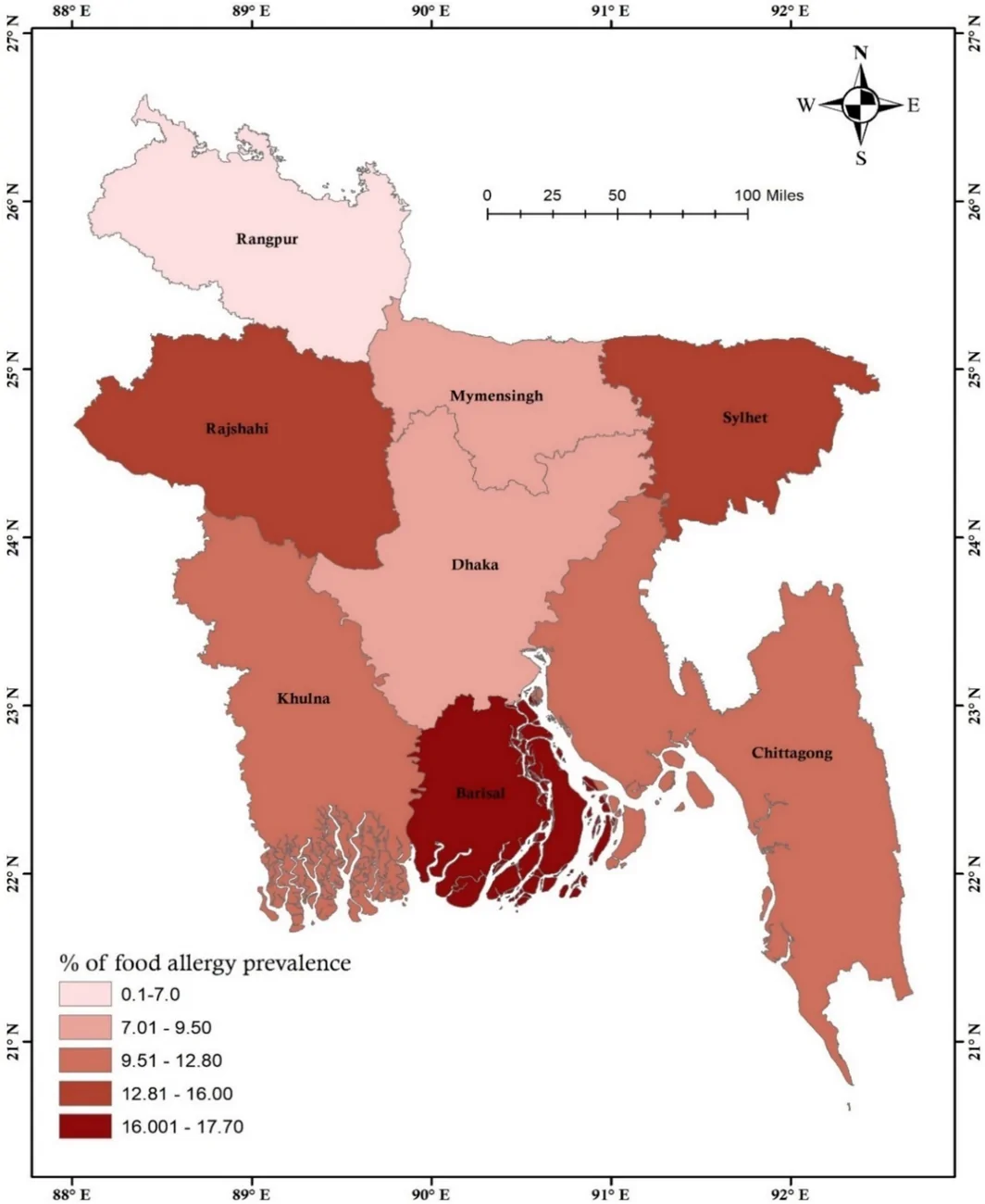
Prevalence of study defined food allergy.

**FIGURE 4:**
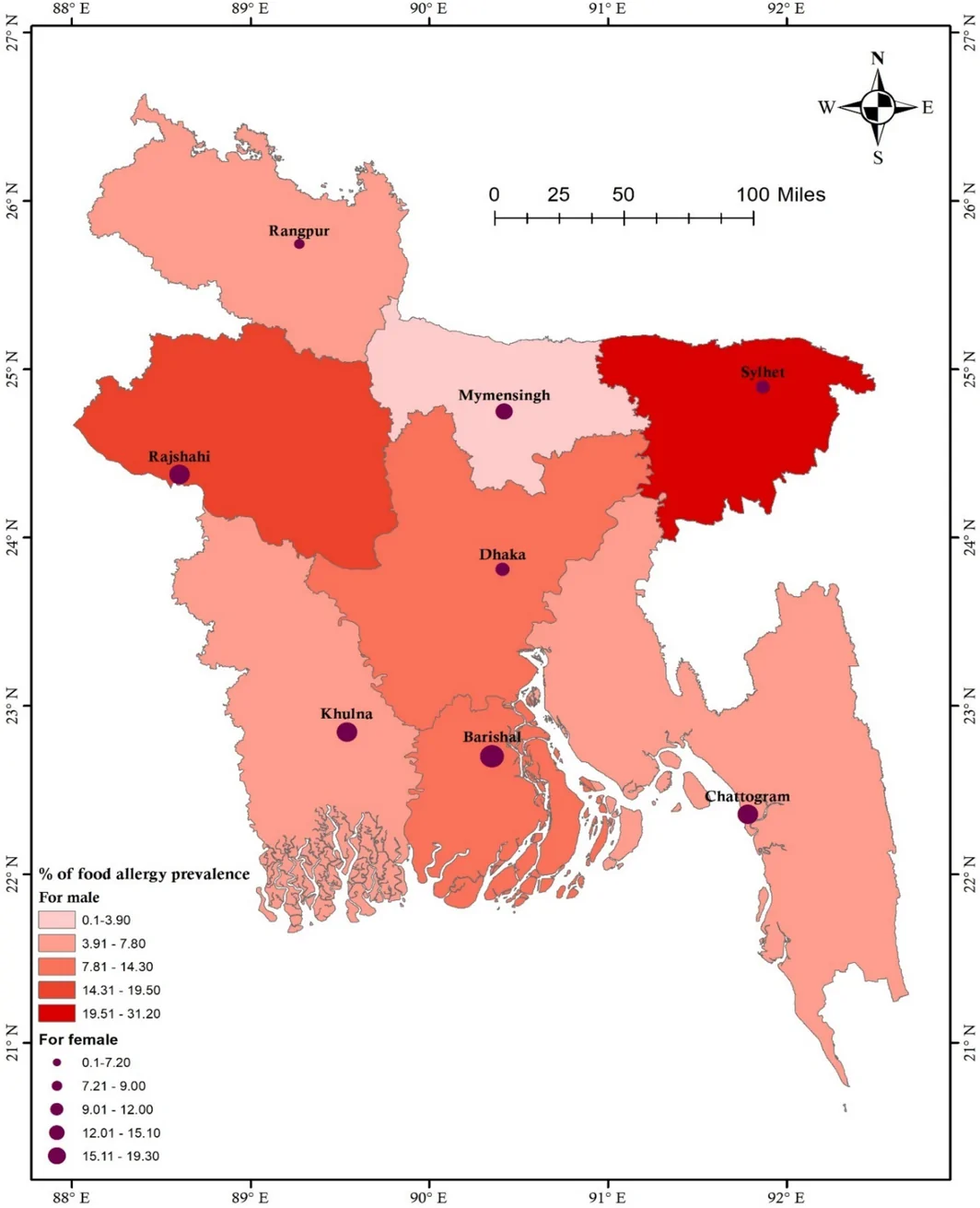
Gender based distribution of food allergy prevalence.

**FIGURE 5:**
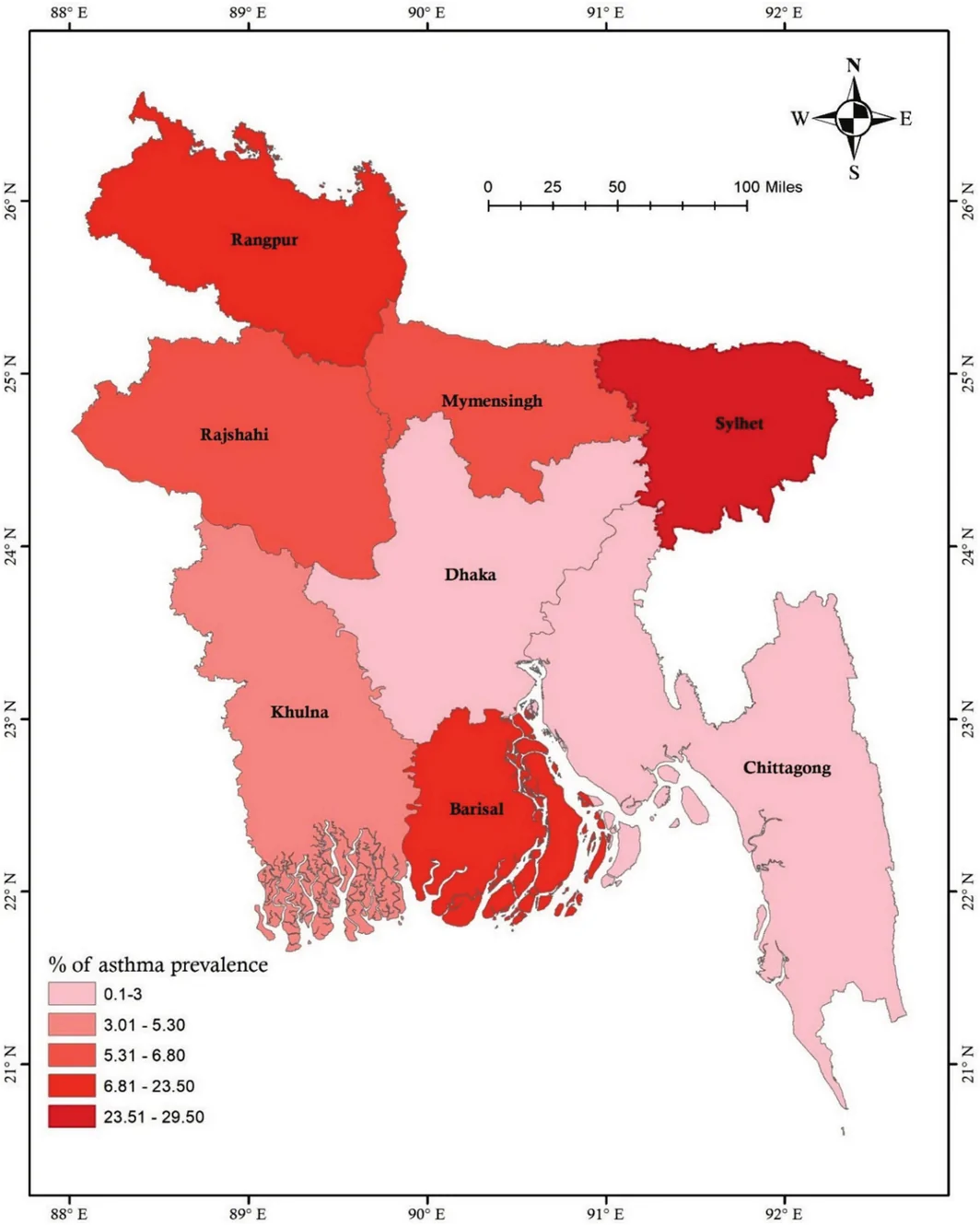
Prevalence of current asthma.

**FIGURE 6:**
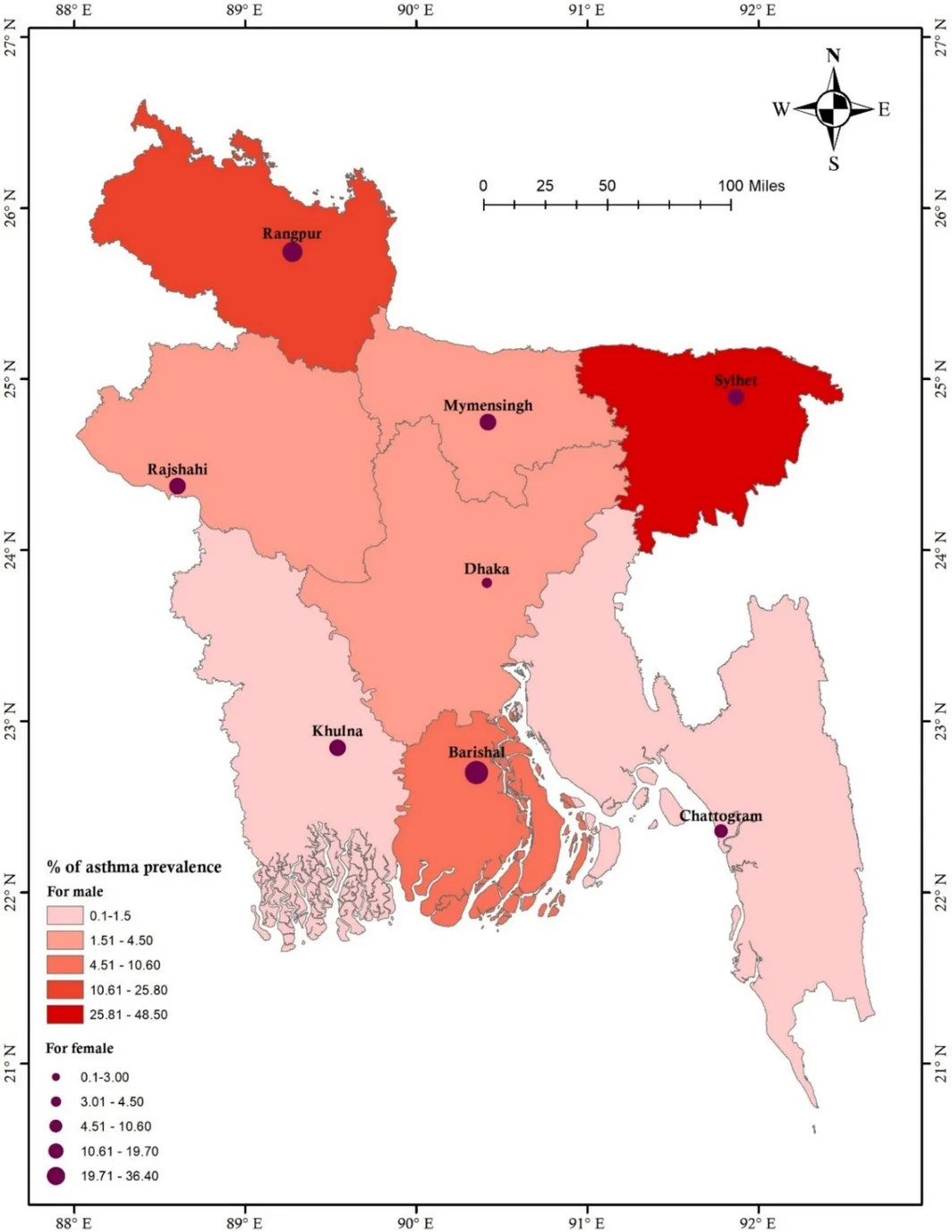
Gender based prevalence percentage of current asthma.

**FIGURE 7:**
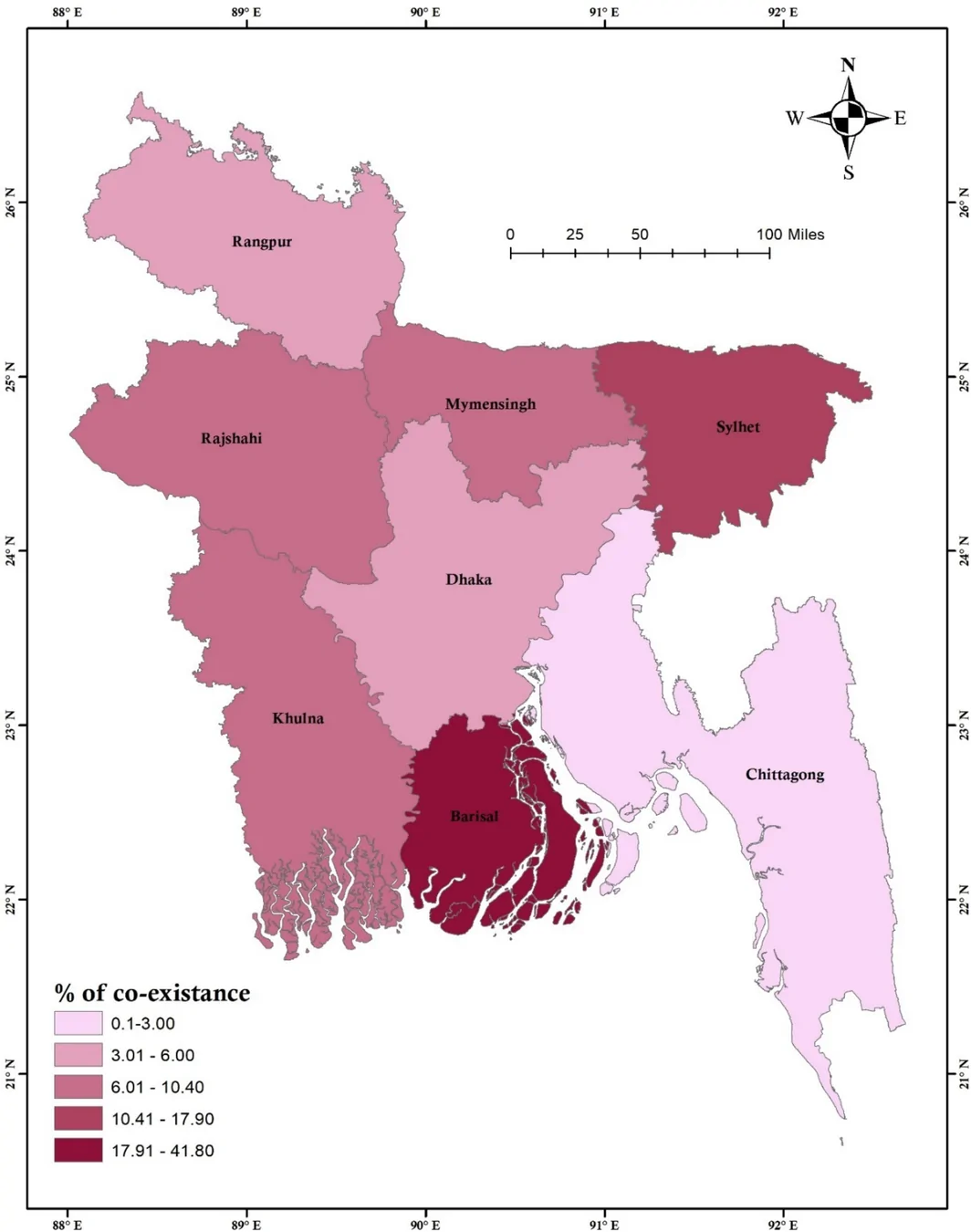
Distribution of coexistence of study defined food allergy and current asthma.

The division‐wise distributions of study‐defined food allergies, current asthma, and their coexistence are presented in Figures 3, 4, 5, 6, 7. Division‐wise variations in the spatial distribution of study‐defined food allergies were statistically significant (*χ* ^2^ = 32.734, *p* < 0.001). The prevalence of study‐defined food allergies was higher in the Barisal, Sylhet, and Rajshahi divisions than in the Rangpur division (Figure 3). The sex‐based distribution of food allergies across divisions revealed that the prevalence of study‐defined food allergies varied significantly across divisions among females (*χ* ^2^ = 40.146, *p* < 0.001) but not among males (*χ* ^2^ = 11.598, *p* = 0.115). Male participants from Sylhet and Rajshahi and female participants from Barisal, Khulna, Chattogram, and Rajshahi had the highest prevalence of study‐defined food allergies. The Mymensingh division had the lowest prevalence of food allergies among males, whereas the Rangpur division had the lowest prevalence among females (Figure 4).

### 3.9 GIS‐Based Distribution of Current Asthma Across Divisions

Division‐wise variations in the spatial distribution of current asthma were significant (*χ* ^2^ = 88.42, *p* ≤ 0.001). Current asthma was most prevalent in the Barisal, Sylhet, and Rangpur divisions, whereas the lowest prevalence was observed in the Dhaka and Chattogram divisions (Figure 5). The gender‐based distribution of current asthma varied significantly across divisions for both males (*χ* ^2^ = 54.495, *p* < 0.001) and females (*χ* ^2^ = 58.878, *p* < 0.001). The prevalence of sex‐based current asthma was highest in the Rangpur and Sylhet divisions for males, whereas for females, it was highest in the Barisal and Rangpur divisions. The Dhaka division had the lowest prevalence of current asthma among female participants, whereas the Chattogram and Khulna divisions had the lowest prevalence among male participants (Figure 6).

### 3.10 GIS‐Based Distribution of Coexistence of Study‐Defined Food Allergy and Current Asthma

Division‐wise variations in the spatial distribution of coexistence of food allergies and current asthma were statistically significant (*χ* ^2^ = 69.783, *p* < 0.001). defined food allergies and current asthma as coexisting conditions, mostly in the Barisal and Sylhet divisions among schoolchildren. The Chattogram division had the lowest prevalence of coexistence of study‐defined food allergies and current asthma (Figure 7).

## 4 Discussion

This study is the first to thoroughly measure the prevalence of food allergies among Bangladeshi schoolchildren based on a convincing clinical history. We also investigated the relationship between study‐defined food allergies and several risk factors, as well as its interplay with the coexistence and severity of asthma. Our findings indicate a remarkably high 12‐month prevalence of food allergies (29.3%), which is considerably higher than that reported in similar age groups worldwide. We employed a comprehensive clinical history‐based methodology to ascertain food allergies, emphasizing specific IgE‐mediated reactions, their onset, and the associated food items. This approach provides a more precise distinction between food allergies and non‐IgE‐mediated diseases, such as food intolerance or poisoning. Although oral food consumption challenges (OFC) were not performed, a significantly higher prevalence was obtained with this approach than with OFC or similar history‐based diagnoses. For instance, estimates in Kuwait (4.1%), Australia (4.5%), Lebanon (6%), France (2.1%), Korea (4.06%), the UK (5.3%), and Canada (1.5% for peanut allergy) were notably lower than those found in our study (Ziyab 2019; Pénard‐Morand et al. 2005; Venkataraman et al. 2018; Sasaki et al. 2017; Sakakini et al. 2022; Kim et al. 2017; Kagan et al. 2003). Such discrepancies might result from variations in diagnostic criteria, dietary patterns, environmental exposure, genetic susceptibility, or access to healthcare systems.

Vegetables (76.1%), beef (61.3%), and shellfish (58.0%) were the most frequently reported food allergens. This trend shows that both plant‐ and animal‐derived allergens are predominant, with vegetables unexpectedly dominating the list, which is contrary to global patterns. Another study also identified shellfish as a common food allergen (Ziyab 2019). Nevertheless, our results contradict the findings of that study, which identified eggs and fish as the most common allergens (Ziyab 2019), underlining the geographical and dietary differences in allergen exposure and sensitization in various groups of the population. In another study, animal‐based foods were found to be the most common cause of food allergies in school children (Feng et al. 2022). In our study, beef was the most frequently reported animal‐derived allergen, although vegetables showed the highest overall prevalence among all reported allergens. In line with previous research, cow's milk and dairy items, fruits, vegetables, eggs, and nuts are common food allergens in schoolchildren (Sakakini et al. 2022). This inconsistency suggests that genetic factors, cultural food habits, and food preparation techniques may play a role in the sensitization of allergens. One of the most prevalent IgE‐mediated food allergens in children is peanut allergy, which is prevalent among all children in the United States (1 in 2.5). The most common causative food allergen among single food items was peanuts in a study of schoolchildren (Kim et al. 2017). However, peanuts were one of the least prevalent food allergens reported in our study. The fact that peanut allergies were not prevalent in our study underscores the need for context‐specific studies because regional nutritional habits and early life exposure may shape allergy development and manifestation.

Given this distinctive pattern of vegetable, beef, and shellfish allergies, it is important to consider how sensitization to these foods might influence asthma expression and severity in this population. IgE‐mediated food allergy can promote asthma through shared type 2 inflammatory pathways: allergen uptake at epithelial barriers (gut, skin, or airway) leads to Th2‐skewed responses, IgE production, and mast cell/basophil activation, with release of histamine, leukotrienes, and cytokines that drive bronchial hyperresponsiveness and airway inflammation (Anvari, Miller, Yeh, and Davis 2019; Crespo and Cabanillas 2023; Kanagaratham et al. 2020). Repeated food‐triggered systemic reactions may therefore worsen asthma control and increase the risk of severe exacerbations during anaphylaxis (Chong et al. 2020; Maspero et al. 2022). Airborne exposure to food proteins is another plausible mechanism: inhalation of aerosolized allergens from cooking or food processing (e.g., bell pepper/other vegetables, fish and seafood, wheat flour) has been shown to induce wheezing and “inhalant food” asthma in sensitized individuals (Jeebhay et al. 2019). Shellfish allergens, particularly tropomyosin, are heat‐ and protease‐stable and readily become airborne during cooking, making them potent triggers of respiratory symptoms and severe asthma attacks on inhalation (Lopata et al. 2016). Beef allergy may coexist with sensitization to other animal proteins or lipid‐associated allergens, which can enhance Th2 immunity and airway responses through interactions at the epithelial surface and within airway tissues (Lisiecka 2026). For plant foods such as vegetables, allergenicity is often linked to cross‐reactive proteins (e.g., Bet v 1–related PR‐10 proteins, profilins, lipid transfer proteins), where primary sensitization to aeroallergens (pollen) can drive secondary food reactions and contribute to a broader, more severe polysensitized asthma phenotype (Fernández Rivas 2003). Finally, early food sensitization (especially persistent IgE responses) is a strong predictor of later asthma and multimorbid allergic disease, suggesting that the high rates of vegetable, beef, and shellfish allergy in this cohort may indicate a subgroup with heightened systemic type 2 inflammation and greater asthma severity (Alduraywish et al. 2016).

The most common symptoms observed in affected schoolchildren were dermatological, including itching; stinging or burning of the lips, mouth, or throat; urticaria (hives); and flushing, which is in accordance with the global literature (Ziyab 2019; Sakakini et al. 2022; Feng et al. 2022). These symptoms are caused by the release of histamine via IgE‐mediated mast cell degranulation (Anvari, Miller, Yeh, and Davis 2019). Histamine and other mediators primarily affect the skin, rendering the symptoms visible and immediate. Approximately, 16.9% of schoolchildren experienced respiratory symptoms, such as wheezing, which is also in agreement with the results of another study on schoolchildren (Ziyab 2019). Alarmingly, 22.2% of allergic schoolchildren experienced anaphylaxis, a life‐threatening reaction, much higher than that schoolchildren in Kuwait (3.9%) (Ziyab 2019). Differences in diagnostic criteria or reporting standards may have contributed to this gap.

The results of our study showed a greater prevalence of study‐defined food allergies in female participants, which is consistent with the findings of similar studies reporting a higher prevalence among girls than boys (Ziyab 2019). Furthermore, a large Swedish cohort study that included over one million children also reported that food allergies were more prevalent in female children, which aligns with our results (Mitselou et al. 2018). Sex differences in food allergies can be explained by hormonal and biochemical differences in males and females, and their immune systems in particular. It is well recognized that female sex hormones, particularly estrogen, alter immunological responses in a manner that may increase susceptibility to developing allergic disorders. The Th2 (T‐helper type 2) immunological pathway, which is strongly linked to the emergence of allergic disorders, has been demonstrated to be strengthened by estrogen. It stimulates the activity of mast cells and basophils, which release histamine and other mediators during allergic reactions and encourages B cells to produce IgE (Bonds and Midoro‐Horiuti 2012).

Age was positively associated with study‐defined food allergy, with food allergy being less common among 12–13‐year‐old schoolchildren than among 16–18‐year‐old adolescents. Similar associations between age and food allergy have been reported in previous studies (Branum et al. 2012; Gupta et al. 2011). However, this finding should be interpreted cautiously, as food allergy across childhood and adolescence follows a dynamic pattern, with the resolution of some early‐life allergies and the emergence of new allergies during later childhood and adolescence (Venkataraman et al. 2018; Lee et al. 2024; Peters et al. 2017). Therefore, the observed age difference should not be interpreted as evidence that food allergy simply increases with age. Because of the cross‐sectional design, the association may also reflect cohort effects, differences in early‐life environmental or dietary exposures, or variation in the recognition and reporting of allergic reactions across age groups rather than a direct effect of aging. Furthermore, older adolescents may be better able to recognize and recall previous allergic reactions than younger children, which could have contributed to the observed association.

Another finding of this study was that children with secondary education were twice as likely to have study‐defined food allergy as those with higher secondary education. This association should also be interpreted cautiously, as educational level is unlikely to act independently and may instead serve as a proxy for socioeconomic status, health literacy, healthcare access, or differences in dietary and environmental exposures (Perry et al. 2023; Tepler et al. 2022). Children in different educational groups may also differ in their awareness and reporting of food allergy symptoms or in their likelihood of receiving medical evaluation, potentially influencing the observed prevalence. Therefore, the observed association should not be interpreted as indicating that educational level directly influences food allergy risk. Rather, it may reflect the combined effects of unmeasured socioeconomic and contextual factors. Further longitudinal studies incorporating objective clinical confirmation of food allergy are needed to clarify these relationships.

The results further showed that asthma was strongly correlated with a higher prevalence of food allergies, suggesting a close relationship between respiratory and food‐related allergic conditions in schoolchildren. Conversely, in a different comparable study, study‐defined food allergy did not show a significant correlation with an increased prevalence of asthma (Ziyab 2019). However, another survey‐based study revealed that food allergies are prevalent among school‐aged children with asthma residing in inner‐city neighborhoods (Friedlander et al. 2013), indicating the intersection between the two conditions in some settings. A different study indicated that asthma and food allergies are likely to exacerbate each other in children (Caffarelli et al. 2016). This is especially alarming because asthma can predispose patients to serious or life‐threatening allergic reactions to food consumption. Research has also indicated that individuals with food allergies and asthma are more prone to severe anaphylactic reactions than those with food allergies alone (Kewalramani 2010). It is possible that this elevated risk is caused by shared inflammatory mechanisms in the two diseases, including augmented immunoreactivity and airway hypersensitivity. These results indicate a clinical need for both asthma and food allergies in children in terms of proper management to ensure that their co‐existence does not complicate the treatment program and poses a risk of negative health outcomes. Thus, early diagnosis, treatment, and increased awareness are important for children with either disease.

A strong positive association between study‐defined food allergy and current asthma was observed in the present study, consistent with previous studies showing that children with food allergy have a higher prevalence of asthma and greater asthma morbidity (Schröder et al. 2009; Hossny et al. 2024). Study‐defined food allergy was also associated with more severe asthma manifestations, including frequent wheezing attacks and wheezing that limited speech, which is in agreement with earlier reports (Ziyab 2019). However, the inverse association observed between study‐defined food allergy and wheezing that disturbed sleep contrasts with previous findings and should be interpreted cautiously rather than as evidence against the overall food allergy–asthma relationship. This unexpected finding may reflect residual confounding, the relatively small number of participants within asthma severity subgroups, or the heterogeneous nature of childhood asthma, whereby individual symptom‐based severity indicators may represent different asthma phenotypes rather than a single severity continuum (Pijnenburg et al. 2022; Cucco et al. 2025). In addition, differences in study design, asthma and food allergy definitions, symptom ascertainment, and reliance on questionnaire‐based reporting may have contributed to the discrepancy between our findings and those reported previously (Ziyab 2019). Therefore, the paradoxical association with sleep‐disturbing wheeze should be regarded as an isolated inconsistent finding that requires confirmation in larger longitudinal studies using objective measures of both food allergy and asthma severity. Nevertheless, the overall body of evidence, together with our findings, supports a close relationship between food allergy and asthma, highlighting the importance of food allergy screening and integrated clinical management among children with asthma to reduce morbidity and prevent severe allergic complications.

The rates of food allergies were higher in the Barisal, Sylhet, and Rajshahi divisions, with Barisal recording the highest prevalence compared to other regions, including Dhaka, Rangpur, Mymensingh, Khulna, and Chattogram. Asthma was also prevalent in Barisal, Sylhet, and Rangpur Divisions. It is important to note here that Barisal and Sylhet have been diagnosed as hot spots where food allergies and asthma coexist, indicating that children in these divisions may be exposed to a particularly unfavorable mix of environmental and social determinants. Such clustering may be due to regional variations in genetic susceptibility to atopic disorders or environmental or lifestyle influences on the response of the immune system. Higher population density, traffic‐related and indoor air pollution, damp housing, and lower sunlight exposure have all been implicated in increasing the risk of food allergies and asthma, especially in more urbanized or disadvantaged settings (Levin et al. 2020; Lotfata et al. 2023; Morillo‐Argudo et al. 2020; Song et al. 2023). Rapid nutritional transition toward Westernized, energy‐dense diets rich in fast foods and fried meats, and lower intake of fresh fruits and vegetables, also appear to promote allergic diseases through effects on the microbiome and epithelial barriers, and such dietary shifts are most pronounced in economically developing regions (De Waal et al. 2020; Leung, Pacharn, et al. 2024; Leung, Xing, et al. 2024; Shi et al. 2025; Sozener et al. 2022). In addition, children in lower‐SES areas may face overcrowded housing, higher smoking exposure, limited access to allergy and asthma care, and lower use of preventive services, all of which can amplify both the true burden and the likelihood of diagnosis (Dierick et al. 2020; Jiang et al. 2023; Morillo‐Argudo et al. 2020; Perry et al. 2023; Warren and Bartell 2024). Unlike the other divisions, the Chattogram division was least associated with food allergies and asthma. This might be because of the coastal climate of the area, which probably facilitates better air quality and might reduce common asthma triggers such as air pollution and pollen. Moreover, a local diet consisting of fresh marine dishes, vegetables, and anti‐inflammatory minerals can help prevent allergies and respiratory diseases. The GIS‐based spatial analysis provided additional epidemiological value by identifying geographic heterogeneity in the distribution of study‐defined food allergy, current asthma, and their coexistence across Bangladesh. Such spatial mapping can help identify high‐burden areas, generate hypotheses regarding environmental and contextual determinants of disease, and support geographically targeted public health planning and resource allocation (Murad and Khashoggi 2020; Bentué‐Martínez et al. 2024; Robin et al. 2019). These findings support the need for region‐specific public health strategies, including improving air quality, promoting healthier dietary patterns, and addressing socioeconomic inequities in high‐burden divisions.

### 4.1 Limitations

This study has several limitations. The cross‐sectional design of this study restricted our ability to establish causal relationships among food allergies, asthma, and associated factors. The reliance on self‐reported information without clinical confirmation, such as skin prick testing or serum‐specific IgE assessment, may have introduced recall bias and misclassification, including the possibility of confusing food intolerance or other nonallergic adverse food reactions with true food allergy. These objective diagnostic methods were not feasible because this was a large‐scale, school‐based survey conducted across multiple districts with limited financial, logistical, and laboratory resources. As a result, the reported prevalence may have been either overestimated or underestimated. The direction and magnitude of this potential bias cannot be determined with certainty. In addition to influencing the prevalence estimates, recall and reporting bias may also have affected the observed associations between food allergy, asthma, and other risk factors and should therefore be considered when interpreting the findings. The sample was primarily drawn from rural areas, which may restrict its generalizability, particularly in urban populations. Geographic and institutional diversity were restricted by including only one district per division and two public institutions per district. The sample sizes varied across divisions and were relatively modest for regional comparisons. Consequently, the regional prevalence estimates may have been influenced by small‐area variability, and minor differences between divisions should be interpreted with caution. Furthermore, this study excluded younger children and students attending private schools, and certain critical background information was gathered through parental recall, which may have introduced inaccuracies. Lastly, exposure to the environment and dietary patterns has not been investigated, and allergic symptoms or asthma have not been explicitly assessed. None of the pre‐primary, primary, or under‐or equal to 12‐year‐old children were part of this study. This limitation does not allow us to know more about the burden of food allergies and the risk factors in all age groups.

### 4.2 Practical Implications and Future Directions

The prevalence of food allergies and asthma in this study illustrates the need for specific interventions in the school environment to educate children, parents, and teachers about food allergies, their management, and their response to an allergic reaction as an emergency. The fact that common allergenic agents include local vegetables, meat, and shellfish underlines the significance of culturally specific food allergy management strategies. There are several social interactions that older children can encounter and be exposed to allergies, highlighting the need to focus on age‐specific instructional programs (Baek et al. 2022). Moreover, socioeconomic factors, including a lack of family income, can hinder access to proper healthcare and the treatment of allergic diseases (Gonçalves et al. 2025). Therefore, socioeconomic factors (especially reduced family income) require targeted intervention to ensure that disadvantaged people can receive appropriate allergy education and care (Perry et al. 2023). Moreover, it has been shown that females may be associated with increased susceptibility to food allergies and asthma; therefore, sex‐sensitive practices should be used in educational and medical interventions (Pali‐Schöll and Jensen‐Jarolim 2019; Ridolo et al. 2019). Most children with asthma tend to develop food allergies and can experience more severe asthma symptoms due to food allergies, particularly in severe instances of wheezing (Friedlander et al. 2013). Thus, it is essential to design interdisciplinary approaches to management that will treat both disorders simultaneously, so that medical staff members possess the tools and training needed to treat both comorbidities appropriately. GIS mapping predicted hotspots of food allergies and asthma in places such as Barisal and Sylhet, which necessitated a focused population health intervention in those areas.

## 5 Conclusions

This study demonstrates that food allergies and asthma are common among Bangladeshi schoolchildren, with 12‐month prevalences of 29.3% and 15.9%, respectively. Skin symptoms were the most frequently reported reactions, and more than one‐fifth of allergic children experienced anaphylaxis, indicating considerable clinical severity. Food allergies were more prevalent among females, older adolescents, children from lower‐income households, and those with limited awareness of food allergies, with notable regional variations and the highest prevalence was observed in Barisal. Children with asthma had approximately twice the risk of food allergy, and their coexistence was particularly common in Barisal and Sylhet. These findings underscore the need for targeted, context‐specific awareness, prevention, and management strategies involving parents, schools, and policymakers to reduce the impact of food allergy and its interaction with asthma in Bangladeshi schoolchildren.

## Author Contributions

**Nitai Roy:** conceptualization, investigation, writing – original draft, methodology, validation, visualization, writing – review and editing, software, supervision, resources, project administration. **Sultan Mahmud Imran:** methodology, formal analysis, data curation, writing – review and editing. **Abdullah Al Adib:** visualization, writing – review and editing. **Md. Fazle Rabbi:** methodology, writing – review and editing, investigation.

## Funding

The authors have nothing to report.

## Conflicts of Interest

The authors declare no conflicts of interest.

## Data Availability

Data will be made available on reasonable request.
